# Message from the Editor

**Published:** 2020-12-04

**Authors:** Georgy P. Kostyuk

**Affiliations:** Moscow Healthcare Department

**Figure 1:**
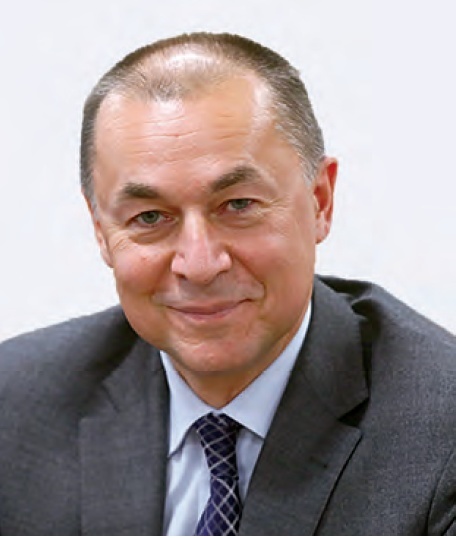
Figure 1. George P. Kostyuk, Editor-in-Chief, Consortium Psychiatricum

DEAR COLLEAGUES,

The second issue of the Consortium Psychiatricum journal is hot off the press! In this issue we focus on both social and clinical topics.

The editorial article raises the importance of the implementation of a syndemic approach in psychiatry. We continue to explore the organization of community psychiatry care in different countries of the world; this time we publish articles from the UK, USA and China.

Two narrative reviews relating to two essential clinical topics - depression in schizophrenia and treatment of negative symptoms in schizophrenia - are written by Russian authors. Works of contemporary experts in this field are cited, along with the works of Soviet psychiatrists that have rarely or never been mentioned previously in this international arena. Therefore, I consider these papers might also be interesting from a historical and a cultural perspective.

The paper containing case reports might also prove interesting from the perspective of the clinical description of patients, observed in Russia. Reiterating the issues surrounding COVID-19, raised in the first issue, we publish the commentary in relation to the new ways that people are having to interact due to the pandemic. The experiences of the destigmatization project that is being undertaken in Moscow, is described in the information section.

We are beginning to collect papers for the thematic issues on the first episode psychosis and ICD-11 implementation aspects, that are scheduled for publication in June and September 2021 respectively. I kindly invite you to submit your manuscripts for these issues.

George P. Kostyuk,

Editor-in-Chief, Consortium Psychiatricum

